# Residual effects of composted sewage sludge on nitrogen cycling and plant metabolism in a no-till common bean-palisade grass-soybean rotation

**DOI:** 10.3389/fpls.2023.1281670

**Published:** 2023-10-20

**Authors:** Mariana Bocchi da Silva, Liliane Santos de Camargos, Marcelo Carvalho Minhoto Teixeira Filho, Lucas Anjos Souza, Aline Renée Coscione, José Lavres, Cassio Hamilton Abreu-Junior, Zhenli He, Fengliang Zhao, Arun Dilipkumar Jani, Gian Franco Capra, Thiago Assis Rodrigues Nogueira

**Affiliations:** ^1^ Department of Plant Protection, Rural Engineering, and Soils, São Paulo State University, Ilha Solteira, SP, Brazil; ^2^ Instituto Federal de Educação, Ciência e Tecnologia Goiano, Rio Verde, GO, Brazil; ^3^ Center of Soils and Environmental Resources of the Campinas Agronomic Institute, Campinas, SP, Brazil; ^4^ Center for Nuclear Energy in Agriculture, Universidade de São Paulo, Piracicaba, SP, Brazil; ^5^ Indian River Research and Education Center, Institute of Food and Agricultural Sciences, University of Florida, Fort Pierce, FL, United States; ^6^ Environment and Plant Protection Institute, Chinese Academy of Tropical Agricultural Sciences, Haikou, China; ^7^ Department of Biology and Chemistry, California State University, Monterey Bay, Seaside, CA, United States; ^8^ Dipartimento di Architettura, Design e Urbanistica, Università Degli Studi di Sassari, Sassari, Italy; ^9^ Desertification Research Centre, Università Degli Studi di Sassari, Sassari, Italy; ^10^ Department of Agricultural Sciences, School of Agricultural and Veterinarian Sciences, São Paulo State University, Jaboticabal, SP, Brazil

**Keywords:** biological N_2_ fixation, organic fertilizer, cover crops, urban waste, no-till

## Abstract

**Introduction and aims:**

In the context of increasing population and decreasing soil fertility, food security is one of humanity’s greatest challenges. Large amounts of waste, such as sewage sludge, are produced annually, with their final disposal causing environmental pollution and hazards to human health. Sludge has high amounts of nitrogen (N), and, when safely recycled by applying it into the soil as composted sewage sludge (CSS), its residual effect may provide gradual N release to crops. A field study was conducted in the Brazilian *Cerrado*. The aims were to investigate the residual effect of successive applications of CSS as a source of N in the common bean (*Phaseolus vulgaris* L. cv. BRS Estilo)-palisade grass (*Urochloa brizantha* (A.Rich.) R.D. Webster)-soybean (*Glycine max* L.) rotation under no-tillage. Additionally, N cycling was monitored through changes in N metabolism; the efficiency of biological N_2_ fixation (BNF) and its implications for plant nutrition, development, and productivity, was also assessed.

**Methods:**

The experiment consisted of a randomized complete block design comparing four CSS rates (10, 15, 20, and 25 Mg ha^-1^, wet basis) to a control treatment (without adding mineral or organic fertilizer) over two crop years. Multiple plant and soil analyses (plant development and crop yield, Falker chlorophyll index (FCI), enzymatic, biochemical, ^15^N natural abundance, was evaluated, root and shoot N accumulation, etc.) were evaluated.

**Results and discussion:**

Results showed that CSS: *i*) maintained adequate N levels for all crops, increasing their productivity; *ii*) promoted efficient BNF, due to the stability of ureide metabolism in plants and increased protein content; *iii*) increased the nitrate content and the nitrate reductase activity in soybean; *iv*) affected urease activity and ammonium content due to changes in the plant’s urea metabolism; *v*) increased N accumulation in the aerial part of palisade grass. Composted sewage sludge can be used as an alternative source to meet crops’ N requirements, promoting productivity gains and N cycling through forage and improving N metabolism.

## 1 Introduction

The use of sewage sludge in agriculture is a viable option for some producers because of the low operating cost associated with its disposal by sewage treatment plants (STPs). It is used as organic fertilizer and/or soil conditioner (Brasil, 2020a), whereas composting sewage sludge has a cost of 49% lower than the cost of disposal in landfills and a profitability of 61% in Brazil (D’avila et al., 2019; Martins et al., 2021). This practice is considered environmentally sustainable and economically viable compared to landfill disposal (Kacprzak et al., 2017). In addition, it provides organic matter and nutrients such as nitrogen, phosphorus, and micronutrients (Nascimento et al., 2020), improving soil fertility (Ferraz et al., 2016) and consequently the productivity of agricultural and forestry crops (Nogueira et al., 2013; Zuba Junio et al., 2019; Abreu-Junior et al., 2020; Elsalam et al., 2021). However, SS may contain heavy metals (Nascimento et al., 2020), organic compounds (Alvarenga et al., 2017), and pathogenic organisms (Murray et al., 2019). In Brazil, the National Council for the Environment (CONAMA) regulated Resolution No. 498, which sets out criteria and procedures for using SS and treatment methods to reduce contaminants in this byproduct (Brasil, 2020a).

Composting has been used to stabilize organic matter and reduce the risks of heavy metals and pathogens, mainly aiming for continuous agricultural sludge recycling (Hargreaves et al., 2008; Wang et al., 2017a). Composted sewage sludge (CSS) as organic fertilizer can improve the chemical, physical, and biological properties of soil, in addition to preventing the contamination and degradation of water resources (Jakubus and Graczyk, 2020; Prates et al., 2020; Prates et al., 2022; Silva et al., 2022a, Silva et al., 2022b). Unlike SS, CSS is already considered a safe product for use in agriculture as it complies with Brazilian standards for registration of organic fertilizers (Brasil, 2020b), which establish maximum limits for heavy metals and pathogenic organisms in CCS. Thus, CSS can be used to fertilize different crops without risks to the environment and human health.

Taking the nitrogen (N) nutrition of the crops into consideration, the amounts of sludge to be applied must meet the N needs of the crop and avoid the generation of nitrate in excessive amounts that will leach in the soil profile to groundwater (Gangbazo et al., 1995). Additionally, SS application provides other nutrients (Boeira et al., 2002). Biological N_2_ fixation (BNF) plays an important role in plant cultivation and mineral fertilization management because it is the most productive and economical N acquisition process and is environmentally viable (Biswas and Gresshoff, 2014).

The supply of CSS as organic fertilizer may at least partially replace the amounts of nutrients applied via mineral fertilizer because the slow and gradual release of the nutrients contained in the compost may result in better use of these nutrients by the plants. The increase in organic matter through the application of CSS may also be highly favorable to the maintenance and improvement of soil quality (e.g., C stock, increased biota, greater moisture retention). In addition, there are still no studies related to the residual effect of CSS on N availability in the soil and on N metabolism through changes in the nodulation, BNF, physiology, and development of soybean and common bean cultivated under a no-tillage system (NTS) in the *Cerrado* region. It is essential to evaluate these aspects to understand the effects caused by the supply of N via CSS and to reduce the use of N fertilizers. Such aspects are reflected in a lower production cost for farmers in addition to reducing the environmental impacts of producing such inputs and the surplus of mineral fertilizers that can be transferred to water bodies and the atmosphere.

In this scenario, which lacks experimental results on the effects of CSS supply on the development and productivity of these crops, the following hypotheses were tested: *i*) the residual effect of CSS applications in *Cerrado* soil may meet the N demand of common bean, forage, and soybean in rotation under an NTS in the *Cerrado*; *ii*) Palisade grass used as a cover crop promotes greater N cycling and an increase in grain yield; and *iii*) CSS supplies N in the system, decreasing the C/N ratio of palisade grass, which favors N mineralization, and providing greater N availability in the soil, which interferes with the efficiency of the BNF process and consequently with N metabolism in common bean and soybean. The objective of this study was to evaluate the residual effect of successive applications of CSS as an N source in a common bean-palisade grass-soybean rotation under NTS in the *Cerrado*, monitoring N cycling through changes in N metabolism (with emphasis on the efficiency of the BNF process), and examining its implications on nutrition, development, and productivity of crops.

## 2 Materials and methods

### 2.1 Experimental area

**Figure 1:**
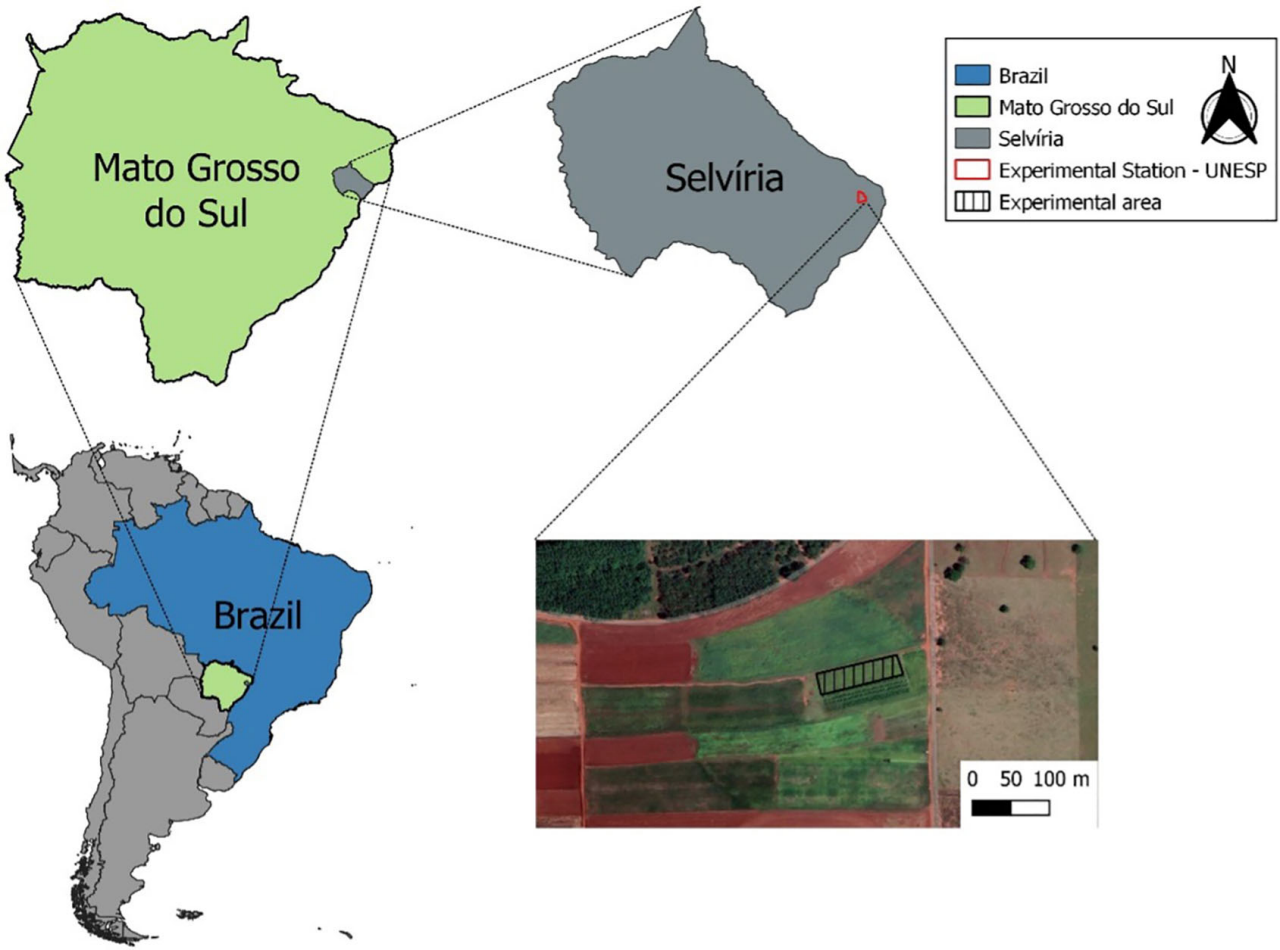
Study location: Selvíria municipality, Mato Grosso do Sul, Brazil.

**Figure 2:**
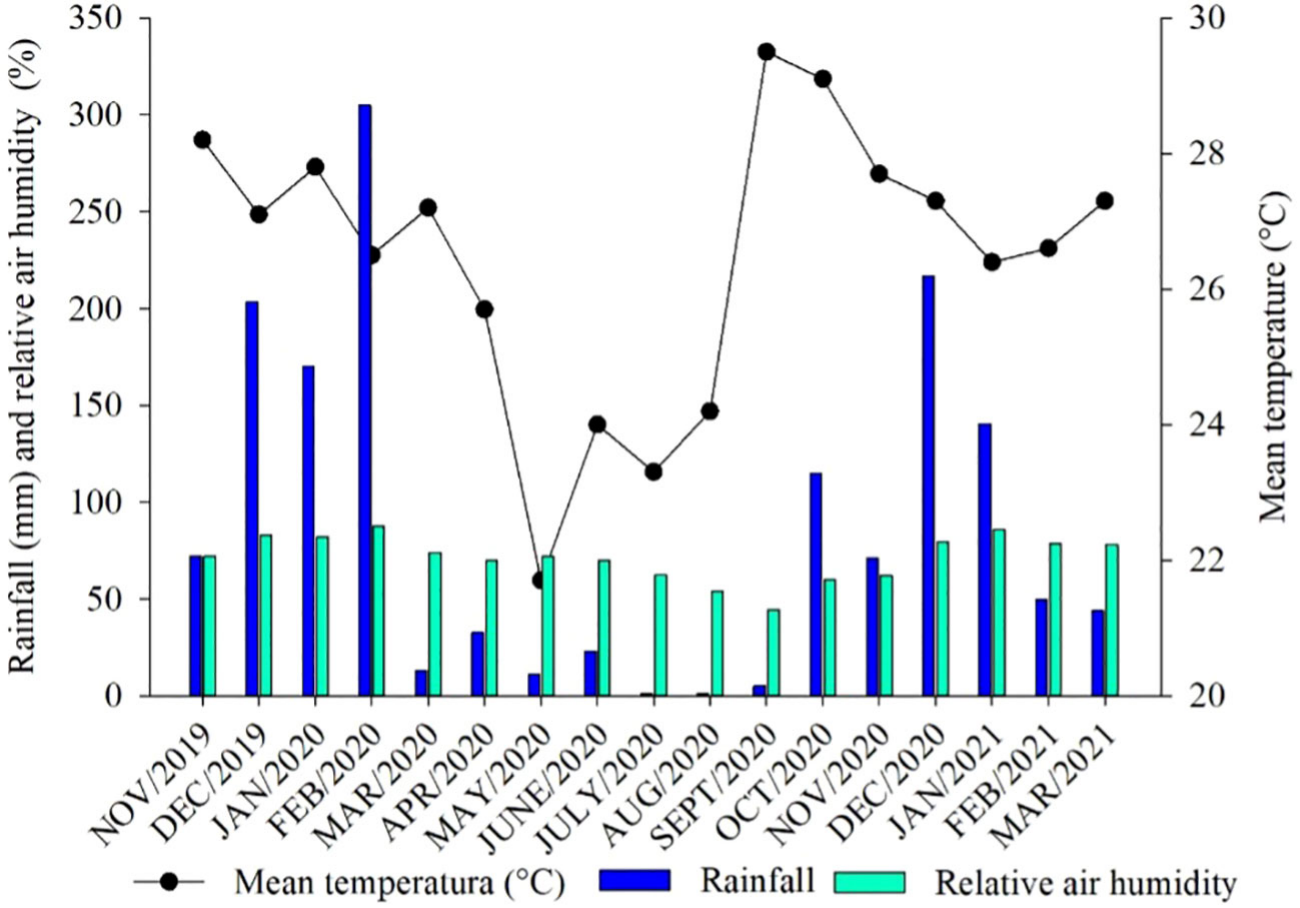
Mean temperature, rainfall, and relative air humidity during the experiment. Data were collected from the weather station of the School of Engineering of Ilha Solteira, São Paulo, Brazil.

The study was conducted under field conditions in the municipality of Selvíria, MS (20°20’35” S and 51°24’04” W, with an altitude of 358 m, **Figure 1**). The region has an annual average rainfall of 1,370 mm, an annual average temperature of 24.5°C, and an average annual relative humidity of 75% (Climate Change Knowledge Portal, 2021). The climate type of the region is Aw, according to the Köppen classification, characterized by rainy summers and dry winters (Lombardi Neto and Drugowich, 1994). During the entire experiment, daily data on temperature, relative air humidity, and rainfall were collected (**Figure 2**).

After a detailed survey, the soil at the site was classified as Rhodic Hapludox, a typical dystrophic sandy clay (Soil Survey Staff, 2014). Before the experiment, samples were collected in the 0–0.2 m layer to evaluate physical (Teixeira et al., 2017) and chemical (Raij et al., 2001) attributes and the following values were obtained: pH (CaCl_2_) = 4.5 ± 0.1; organic matter (OM) = 19.0 ± 1.2 g dm^-3^; phosphorus (P) (resin) = 16 ± 0.6. mg dm^-3^; potassium (K) = 1.7 ± 0.2 mmol_c_ dm^-3^; calcium (Ca) = 13 ± 0.6 mmol_c_ dm^-3^; magnesium (Mg) = 12 ± 1.0 mmol_c_ dm^-3^; potential acidity [hydrogen (H) + aluminum (Al)] = 37 ± 2.3 mmol_c_ dm^-3^; sum of bases (SB) = 27.0 ± 1.7 mmol_c_ dm^-3^; cation exchange capacity (CEC) at pH 7.0 = 63.7 ± 0.9 mmol_c_ dm^-3^; base saturation (V) = 42 ± 3.0%; boron (B) = 0.22 ± 0.04 mg dm^-3^; copper (Cu) = 1.8 ± 0.1 mg dm^-3^; iron (Fe) = 15 ± 0.6 mg dm^-3^; manganese (Mn) = 18.8 ± 0.6 mg dm^-3^; zinc (Zn) = 0.6 ± 0.1 mg dm^-3^; aluminum (Al) = 4.0 ± 0.0 mmol_c_ dm^-3^; clay = 370 ± 19 g kg^-1^, silt = 80 ± 3 g kg^-1^, and sand = 550 ± 13 g kg^-1^.

### 2.2 Experimental design and treatments

A randomized complete block design was adopted, with five treatments and four replications, totaling 20 experimental units. The treatments consisted of the residual effect of four rates of composted sewage sludge - CSS (10, 15, 20, and 25 Mg ha^-1^, wet basis), accumulated from two consecutive applications during the 2017/18 and 2018/19 seasons, and one control treatment (without the application of CSS and mineral fertilizers). To evaluate the residual effect of the two applications of CSS as the N source, no fertilization (mineral or organic) was included during the 2019/20 and 2020/21 seasons.

### 2.3 CSS source and characterization

The CSS used is classified as a Class B organic fertilizer (Brasil, 2020b) and is derived from thermophilic composting of urban organic wastes. In addition to sewage sludge, sludge from wastewater treatment systems generated in agroindustries, such as breweries and various food industries, fruit and vegetable processing, and the remains of industrialized and unserviceable food products make up the sewage sludge. The CSS was characterized according to Conama Resolution No 498 (**Table 1**).

**Table 1:** Chemical and microbiological composition of composted sewage sludge samples.

Characteristic	Unit	2017/18	2018/19	Allowed value ^(1)^
*Chemistry*		^_________^ Dry basis ^________^		
pH _(CaCl2)_	–	7.0 ± 0.1	7.3 ± 0.1	–**^(2)^**
Moisture (60-65 °C)	%	41.0 ± 0.3	34.4 ± 0.5	–
Total moisture	%	45.5 ± 0.2	35.8 ± 0.6	–
Total OM (combustion)	g kg^-1^	308.7 ± 10.0	255.0 ± 7.4	–
CEC	mmol_c_ dm ^-3^	520 ± 20.00	–	–
C/N	–	12.0 ± 0.8	9.0 ± 0.6	–
Total N	g kg^-1^	13.9 ± 0.3	15.3 ± 1.5	–
Total P	g kg^-1^	12.3 ± 1.4	14.1 ± 0.0	–
Total S	g kg^−1^	4.8 ± 0.3	8.4 ± 1.4	–
Na	mg kg^−1^	3930.0 ± 32.0	3915.0 ± 32.0	–
K	g kg^−1^	6.0 ± 2.2	8.2 ± 0.4	–
Ca	g kg^−1^	19.4 ± 4.4	31.1 ± 1.1	–
Mg	g kg^−1^	5.2 ± 0.5	9.9 ± 0.2	–
As	mg kg^−1^	3.2 ± 1.8	–	20.0
B	mg kg^−1^	94.0 ± 4.5	94.0 ± 4.6	–
Cd	mg kg^−1^	1.0 ± 0.1	–	3.0
Cu	mg kg^−1^	237.0 ± 16.5	191.2 ± 5.8	–
Pb	mg kg^−1^	18.1 ± 1.6	–	150.0
Cr	mg kg^−1^	54.3 ± 1.8	–	2.0
Fe	mg kg^−1^	16,400 ± 1300	14,708 ± 249	–
Mn	mg kg^−1^	246.0 ± 37.0	310.0 ± 15.0	–
Hg	mg kg^−1^	0.22 ± 0.1	–	1.0
Mo	mg kg^−1^	5.26 ± 0.2	–	–
Ni	mg kg^−1^	26.5 ± 0.5	–	70.0
Zn	mg kg^−1^	456 ± 8	684 ± 7	–
*Microbiological*				
*Salmonella* sp.	MPN 10 g^−1^	Absent	Absent	
*Thermotolerant coliforms*	MPN g^−1^	0	<103 MPN g^−1^ on dry weight	
Viable helminth eggs	Eggs g^−1^ on dry weight	0.12	<10 Eggs g^−1^ on dry weight	

^(1)^Limits to organic fertilizers use established by the Ministry of Agriculture, Livestock and Food Supply in Brazil (MAPA, 2016). ^(2)^Not determined. MPN, Most probable number; CEC, cation exchange capacity. (mean ± standard deviation; *n* = 3).

According to the total N present in the CSS and the rates used in the experiment, the total amounts of N added to the soil after the two CSS applications were calculated (dry basis): 10 Mg ha^-1^ = 91 kg of N; 15 Mg ha^-1^ = 130 kg of N; 20 Mg ha^-1^ = 173 kg of N; and 25 Mg ha^-1^ = 216 kg of N.

### 2.4 Experiment installation and development

The experimental area was chisel plowed in September 2017 to 0.3 m depth. Lime was incorporated at 2.2 Mg ha^-1^ followed by surface application gypsum at 1.8 Mg ha^-1^ based on recommendations by Raij et al. (1997). One week before soybean planting (November 2017 and 2018), CSS was manually spread out on the soil surface within each plot but not incorporated, considering the moisture content of the material (45% for the first season and 36% for the second season). To evaluate the residual effect of CSS on the development of common bean (2019/20) and soybean (2020/21), no other fertilizer was used for these crops.

As test plants, in the 2017/18 and 2018/19 crop years, soybean in the first crop and corn (*Zea mays* L.) in the second crop were cultivated in succession. In the 2019/20 crop year, a no-tillage system was implemented to cultivate marandu palisadegrass (*Urochloa brizantha* (A.Rich.) R.D. Webster) (as a cover crop). Soon after, the common bean was cultivated (*Phaseolus vulgaris* L. cv. BRS Estilo). In the 2020/21 crop year, marandu palisadegrass was used again as a cover crop, followed by soybean (*Glycine max* L. cv TMG 7063 Ipro).

Each experimental unit consisted of ten rows for marandu palisadegrass (spaced 0.34 m apart) and seven rows of beans and soybeans (spaced 0.45 m apart), with a length of 10 meters, totaling 35 m^2^ per plot and 700 m^2^ of total area. The area of the plot for data collection consisted of three central rows, with 2.5 m from each end eliminated as border.

Marandu palisadegrass was planted in November 2019, with 140 days of cultivation, and then in August 2020, with another 60 days of cultivation. Before the cultivation of each main crop, the marandu palisadegrass was desiccated by glyphosate (1,800 g ha^-1^ of the a.i.) and 2,4-D (670 g ha^-1^ of the a.i.). Common bean and soybean seed were treated with the fungicides, thiophanate methyl + pyraclostrobin (45 g + 5 g a.i. per 100 kg of seed) and fipronil insecticide (50 g a.i. per 100 kg of seed) and with CoMo (200 mL ha^-1^).

Common bean seed was inoculated with *Rhizobium tropici* (Semia 4080, 100 g per 25 kg of seeds). Soybean seed was inoculated with *Bradyrhizobium elkanii* (CEPA SEMIA 5019) and *Bradyrhizobium japonicum* (CEPA SEMIA 5079), following the manufacturer’s recommendations (100 mL for 50 kg of seeds - Masterfix L. Stoller inoculant). Common bean was planted in April 2020, and soybean was sown in November 2020. Irrigation ranging from 10–14 mm per event was applied via a central pivot.

### 2.5 Soil-plant analysis and evaluated parameters

#### 2.5.1 Plants’ nutritional status

Common bean and soybean nutritional analyses were conducted according to the recommendations described by Ambrosano et al. (1997). During common bean’s full flowering period, the third leaf with petiole was randomly collected from the middle third of 10 plants per plot. For soybean, also at full flowering, the third fully developed leaf with petiole was randomly collected from the apex to the base, from 30 plants per plot. The samples were dried in an oven with forced air circulation at 65°C for 72 h, crushed in a Wiley-type mill, and stored until the time of analysis. Leaf N concentration was extracted by sulfuric digestion and determined by the Kjeldahl method (Malavolta et al., 1997).

#### 2.5.2 Falker chlorophyll index (FCI)

The FCI was evaluated at the R6 bean stage, the relative chlorophyll content was evaluated in 10 leaves per plot, with readings performed next to the midrib of the fully expanded leaves (Barbiere Junior et al., 2012). In soybean, at the R2 stage, the readings were taken on the third fully developed trifoliate leaf from the plant’s apex, with an average of 10 readings per leaflet, in five plants per plot. ClorofiLOG portable equipment, model CFL 1030, Falker brand, was used.

#### 2.5.3 Biological N_2_ fixation

Biological N_2_ fixation was evaluated using the ^15^N natural abundance method. Briefly, N_2_ from air contains about 0.3663% ^15^N and the rest (99.6337%) is ^14^N (Boddey et al., 2001). Each unit of delta ^15^N is considered to have natural abundance divided by one thousand, i.e., 0.0003663 atom % excess ^15^N (Cadisch et al., 2000; Lavres et al., 2016). Species capable of obtaining most of the N needed for their nutrition will have δ^15^N values very close to zero, because most of the N will come from the air, which is the standard of the technique and contains 0.3663% ^15^N, meaning zero excess units of δ^15^N (Cadisch et al., 2000; Boddey et al., 2001; Lavres et al., 2016). Conversely, non-N-fixing species (control plants) grown in the same soil will have higher δ^15^N values, close to those of the soil, because all or most of the N required for their development will be derived from the soil. Like other isotopic techniques, this one depends on the basic assumption that fixing and non-fixing plants, grown in the same soil, take up N with the same isotopic labeling from the very close soil volume by both roots (Boddey et al., 2001; Guimaraes et al., 2008; Lavres et al., 2016). Sub-samples of dried and ground material from diagnostic leaf and grain of common bean and soybean were analyzed for % N and δ^15^N on an automated mass spectrometer coupled to an ANCA-GSL N analyzer (Sercon Co., UK). The proportion of N in plants that can fix N_2_ from the air by the BNF process was calculated by the equation of Shearer and Kohl (1986):


(1) $$\%\text{BNF}=100\times ({\text{δ}}^{15}\text{N}\;\text{reference}-{\text{δ}}^{15}\text{N}\;\text{fixing}\;\text{plant})/{\text{δ}}^{15}\text{N}\;\text{reference}-\text{B}$$

where:

% BNF = percentage of N obtained from BNF in the fixing plants;
δ^15^ N reference = natural abundance of ^15^N in the reference (non-N-fixing) plant;
δ^15^ N fixing plant = natural abundance of ^15^N in common bean and soybean plants;
B = fractional contribution of ^15^N relative to ^14^N by the fixing plants in soil N uptake. For common bean, the ‘B’ value used in the present study was -1.2 ‰ determined for the common bean cultivars grown on an N-free hydroaeroponic culture fully dependent on BNF (Pacheco et al., 2017), and -1.17‰ for soybean (Guimaraes et al., 2008).

#### 2.5.4 Total soil N and C

Soil sampling (0-0.1 m and 0.1-0.2 m depth) was performed at the end of each crop cycle, within the useful area of each plot. Five sub-samples were randomly collected per plot to compose a sample. These samples were taken with the help of a soil sampler and, afterwards, air dried, crushed and passed through a sieve with 2 mm of mesh opening, packed in polyethylene bags, identified, and stored in a dry chamber until the moment of the analyses. The total C and total N contents were determined using an automatic elemental analyzer (Swift, 1996). The N-organic content was calculated from the difference between total N and N-mineral (NO_3_^-^ + NH_4_^+^).

#### 2.5.5 Dry matter and nodulation

At the R5 stage of common bean and at the R2 stage of soybean, six plants were collected within each experimental plot with the aid of a cutting shovel. The plants were separated into shoots and roots. All excess soil from the roots was removed with water and a sieve to avoid losses. The nodules were removed from the roots, counted, and then passed through a 2 mm sieve, and those larger than 2 mm were cut in half to observe their viability (nodules of normal appearance with the presence of leghemoglobin, with a characteristic reddish color). After this last stage, the nodules, roots, and shoots were packed in paper bags and placed in a forced air oven at 65°C for 72 h. The following were evaluated: root and shoot dry matter, the number of total nodules, the number of viable nodules, and the nodule dry matter (Matoso and Kusdra, 2014).

#### 2.5.6 Root and shoot N accumulation

After drying and weighing, the roots and shoots from the previous stage were ground in a Wiley-type mill with a 40-mesh sieve and subjected to sulfuric digestion and steam distillation to determine the N concentrations (Malavolta et al., 1997). The accumulated amounts of N were calculated based on the N concentrations and dry matter production.

#### 2.5.7 Plant development and crop yield

At physiological maturity of common bean (R9; ~ 80 DAE) and soybean (R8; 100 DAE), 10 random plants were collected from the useful area of each plot to analyze yield parameters. The following were analyzed for common bean: 100-grain weight, number of grains per plant, number of grains per pod, number of pods per plant, and pod length. For soybeans, the following were analyzed: 100-grain weight, number of pods per plant, number of grains per pod, plant height, and first pod insertion height. At the end of each crop cycle, all plants in the useful area were harvested and manually threshed to avoid losses. After these procedures, the calculations were performed with extrapolation to kg ha^-1^ and corrected for 13% moisture (wet basis) to estimate crop yield (Sabundjian et al., 2016).

#### 2.5.8 Enzymatic analyses

For the enzymatic analyses, five leaves were collected from each plot at the R6 bean and R2 soybean stages, following the recommendations for the analysis of nutritional contents (Ambrosano et al., 1997). The leaves were placed on ice in a thermal box to preserve enzymatic activity until the time of analysis.

- Urease activity: The *in vivo* samples were prepared by adapting the methodology described by Hogan et al. (1983). The leaf tissue (200 mg of green leaves, cut into “strips” with a width of 1 mm) was placed in a medium containing 8 mL of NaH_2_PO_4_ buffer with urea (12.61 g L^-1^), pH 7.4, and incubated for 3 h at 30°C under constant agitation. In a test tube containing 0.5 mL of the extract obtained after incubation, 2.5 mL of reagent I (1.25 mL of crystal phenol, 12.5 mg of sodium nitroprusside, and 250 mL of distilled water) and 2.5 mL of reagent II (1.25 g of NaOH, 13.4 g of Na_2_HPO_4_.12H_2_O, 2.5 mL of NaOCl, and 250 mL of distilled water) were added. The tubes were incubated in a water bath at 37°C for 35 min. After incubation, the reaction was measured by colorimetry in a spectrophotometer at 625 nm. Urease activity was measured through the production of N-NH_4_, according to the method by McCullough (1967);
- Nitrate reductase activity: Leaf samples were collected in the morning, and 0.5 g of fresh leaf tissue was cut into thin strips, placed into a test tube containing 5 mL of NaH_2_PO_4_ buffer, pH 7.5, with KNO_3_ and incubated in a water bath at 30°C for 60 min in the dark. Then, 1 mL of the extraction solution, 0.5 mL of 1% sulfanilamide, and 0.5 mL of 0.02% naphthylethylenediamine were added. After this step, the reaction was measured by colorimetry in a spectrophotometer at 540 nm. The assay and the determination of NR activity followed the recommendations described in Radin (1974).

#### 2.5.9 Biochemical analyses

For the biochemical analyses, five leaves were collected from each plot at stage R6 of common bean and R2 of soybean, according to the recommendations for the analysis of nutritional contents (Ambrosano et al., 1997). Initially, soluble compounds were extracted (Bieleski and Turner, 1966), in which 0.5 g of plant material was ground in 5 mL of MCW (600 mL of methanol, 250 mL of chloroform, and 150 mL of distilled water) and homogenized in a centrifuge at 8500 rpm for 15 min. After centrifugation, the supernatant was added to another tube, and the precipitate was used for the protein assay. For every 4 mL of supernatant, 1 mL of chloroform and 1.5 mL of distilled water were added, and the samples were allowed to rest for 24 h in the refrigerator. After this period, the fat-soluble phase was discarded, and the volume of the water-soluble phase was recorded and stored until the time of analysis. After extraction, the following physiological analyses were performed:

- *Protein* quantification: 5 mL of 0.1 M NaOH was added to the precipitate resulting from the extraction of soluble compounds and placed in a centrifuge at 8500 rpm for 15 min. After this period, 5 mL of Bradford reagent was added to 100 µl of supernatant and incubated for 3 min. The reaction was measured by colorimetry in a spectrophotometer at 595 nm (Bradford, 1976).
- Quantification of *amino acids*: The reaction sample consisted of 25 µL of the water-soluble phase + 975 µL of distilled water, 500 µL of pH 5.0 citrate buffer, 200 µL of 5% methyl glycol ninhydrin, and 1 mL of 0.0002 mol L^-1^ KCN and was incubated in a water bath at 100° C for 20 min. Then, the mixture was incubated for 10 min at room temperature, after which 1 mL of 60% ethanol was added. The reaction was measured by colorimetry in a spectrophotometer at 570 nm (Yemm and Cocking, 1955).
- Quantification of *nitrate*: Following the methodology described by Cataldo et al. (1975), we used 50 µL of the water-soluble phase together with 200 µL of 5% salicylic acid in H_2_SO_4_ and waited 20 min at room temperature. Then, 4.75 mL of NaOH 2N was added, and the mixture was cooled to room temperature until the reaction stabilized. Afterward, the assay was measured by colorimetry in a spectrophotometer at 410 nm.
- Quantification of *ammonium*: For the ammonium assay, 100 µL of the water-soluble phase was used, and 500 µL of reagent I (1.25 mL of crystal phenol, 12.5 mg of sodium nitroprusside, and 250 mL of distilled water) and 500 µl of reagent II (1.25 g of NaOH, 13.4 g of Na_2_ HPO_4_.12H_2_O, 2.5 mL of NaOCl and 250 mL of distilled water) were added; the samples were then placed in a water bath at 37°C for 1 h. After this period, the reading was continued by colorimetry in a spectrophotometer at 630 nm (McCullough, 1967).
- Quantification of *ureides*, *allantoic acid*, and *allantoin*: according to the method proposed by Vogels and van der Drift (1970), the assay consists of four stages. Step I: 250 µL of the water-soluble phase was diluted in 500 µL of distilled water, 250 µL of NaOH 0.5N and 1 drop of 0.33% phenylhydrazine were added, and the samples were heated in a water bath at 100°C for 8 min and then cooled to room temperature. Step II: 250 µL of HCl 0.65N was added, and the samples were heated in a water bath at 100°C for 4 min and then cooled to room temperature. Step III: 250 µL of phosphate buffer pH 7.0 and 250 µL of phenylhydrazine were added and left to stand for 5 min at room temperature and 5 min on ice. Step IV: While still on ice, 1.25 mL of 37% HCl (which must be kept in the freezer for analysis) was added; the samples were then removed from the ice, and 250 µL of K_3_Fe(CN)_6_ was added; the tubes were shaken and incubated for 15 min at room temperature prior to reading by colorimetry in a spectrophotometer at 535 nm. To quantify total ureides, we followed the four steps described above. To quantify allantoic acid, we diluted 250 µL of the water-soluble phase in 500 µL of distilled water and followed the assay beginning with step II. To quantify allantoin, we subtracted the value obtained for total ureides from that obtained for allantoic acid.

#### 2.5.10 Marandu palisadegrass phytomass production and N accumulation

To evaluate the biomass production of marandu palisadegrass in the 2019/20 crop, two cuts were performed at 70 and 140 days after emergence (DAE), and in the 2020/21 crop, a cutoff was performed at 60 DAE. The cuts were performed randomly at four points per plot as close to the soil as possible using a 0.25 m^2^ square. After the first cut (crop 2019/20), Triton was applied to uniform the area, and the plants sprouted again to form a new straw on the soil. In the second cut of the 2019/20 crop and in the only cut of the 2020/21 crop, after completion, the grass was desiccated with glyphosate herbicide (1,800 g ha^-1^ of the a.i.) and 2,4-D (670 g ha^-1^ of the a.i.) for crop sowing in succession (beans followed by soybeans). The collected samples were dried in a forced circulation oven at 65°C for 72 h, and the plant material was weighed to obtain the total amount of straw formed (kg ha^-1^) in each season evaluated.

Nitrogen accumulation was evaluated by analyzing the shoot samples of marandu palisadegrass from each crop. In particular, shoots were weighed and then ground in a Wiley-type mill with a 40-mesh sieve and homogenized, then N concentration was obtained using sulfuric digestion and steam distillation (Malavolta et al., 1997). Based on the N concentration and dry matter production, the accumulated amounts of N were calculated and extrapolated to kg ha^-1^.

### 2.6 Statistical analysis

Statistical analysis was performed using R software (R Core Team, 2019) and AgroEstat software (Barbosa and Maldonado, 2015). The results were subjected to the Shapiro–Wilk normality test and the O’Neill and Mathews test of homogeneity of variances at 5%. After meeting the hypotheses of normality, the results were subjected to analysis of variance, and the means were compared using the HSD-Tukey test (*P* < 0.05) in cases in which the F test was significant.

## 3 Results

### 3.1 The FCI, foliar N, dry matter, and N uptake

**Figure 3:**
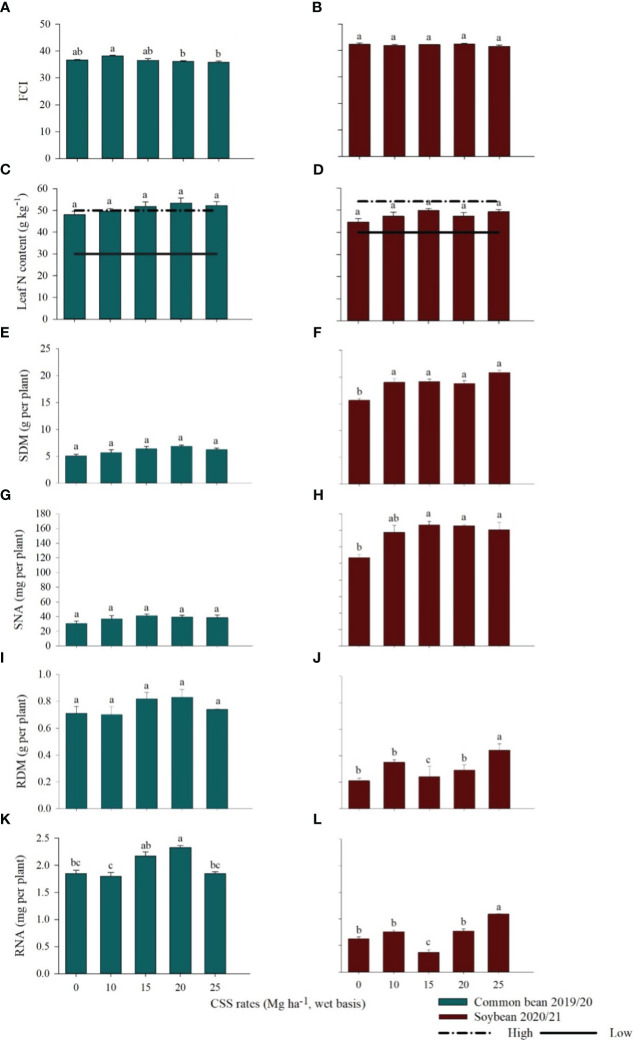
Falker chlorophyll index (FCI) **(A, B)**, leaf N content **(C, D)**, shoot dry matter (SDM) **(E, F)**, shoot N accumulation (SNA) **(G, H)**, root dry matter (RDM) **(I, J)**, and root N accumulation (RNA) **(K, L)** in common bean and soybean plants cultivated under the residual effect of the application of rates of composted sewage sludge (CSS). Means ± standard errors followed by the same letter did not differ from each other by Tukey’s test at 5% probability. The horizontal lines on graph bars represent range of interpretation of N concentrations established by Ambrosano et al. (1997).

The residual effect of CSS influenced the Falker chlorophyll index (FCI) of common bean, with a difference between the studied rates; the highest value of FCI was obtained at the 10 Mg ha^-1^ CSS rate (**Figure 3A**). For soybean, there was no residual effect of CSS on the FCI (**Figure 3B**).

There was no effect of CSS application on N levels in the leaves of common bean and soybean (**Figures 3C, D**, respectively). For common bean, in the control treatment and in the plots that received 10 Mg ha^-1^ CSS, the leaf N concentration of common bean remained within the sufficiency range (30–50 g kg^-1^) established by Ambrosano et al. (1997). However, where the highest rates of CSS were applied, the N levels in the leaves were above the maximum level of N (**Figure 3C**). In soybean, leaf N concentration remained within the range of adequate N concentration (40–54 g kg^-1^) described by Ambrosano et al. (1997), (**Figure 3D**).

Shoot dry matter (SDM), shoot N accumulation (SNA), and the root dry matter (RDM) of common bean were not affected by the residual effect of successive CSS applications (**Figures 3E, G, I**). However, there was greater root N accumulation (RNA) of the common bean plants grown in the plots that received 10 and 15 Mg ha^-1^ of CSS, with accumulated amounts of 1.8 and 2.3 mg per plant, respectively (**Figure 3K**).

In soybean, successive CSS applications had a residual effect on SDM, SNA, RDM, and RNA (**Figures 3F, H, J, L**). The CSS 25 Mg ha^-1^ rate greatly increased SDM, RDM and RNA, i.e., 19.95 g, 0.43 g, and 1.09 mg, respectively. For SNA, the 15 Mg ha^-1^ rate promoted the largest N increase, 146.72 mg per plant.

### 3.2 Biological N fixation (BNF) and total N and C soil

**Figure 4:**
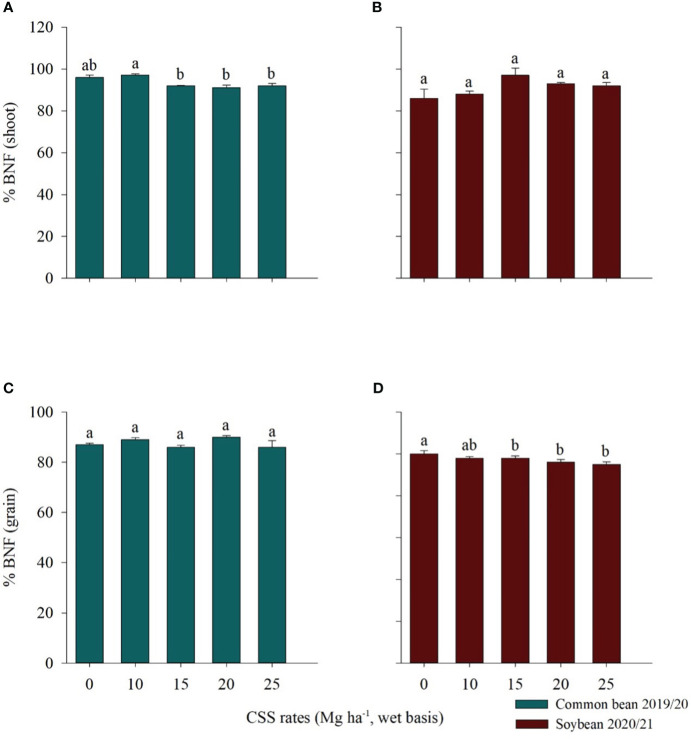
Percentage of BNF (biological nitrogen fixation) in the shoot **(A, B)** and in the grains **(C, D)** of common bean and soybean plants cultivated under the residual effect of the application of rates of composted sewage sludge (CSS). Means ± standard error followed by the same letter do not differ by the Tukey test at 5% probability.

There was a significant influence of the residual effect of CSS on BNF by common bean. The rate of 10 Mg ha^-1^ of CSS and control treatment indicated, respectively, that 97% and 96% of the accumulated N was from BNF, and the rate of 20 Mg ha^-1^ of SSC showed the lowest % of BNF, 91% (**Figure 4A**). There was no difference between the treatments evaluated for soybean, with values varying between 86% and 97%, in the treatments with 0 and 15 Mg ha^-1^ of CSS, respectively (**Figure 4B**).

In grain (phenological stage R9), it was observed that there was no influence of the residual effect of CSS for common bean, where there was a variation of 86% to 90% in the rate of 25 and 20 Mg ha^-1^ of CSS, respectively (**Figure 4C**). For soybeans, on the other hand, the influence of the residual effect of CSS on the grains was noted. The control treatment and the 10 Mg ha^-1^ CSS rate presented the highest % BNF averages, 100 and 98%, respectively, and were similar to each other, while the other rates evaluated (15, 20 and 25 Mg ha^-1^ CSS) the BNF values were, respectively, of 97%, 96% and 95% (**Figure 4D**).

**Figure 5:**
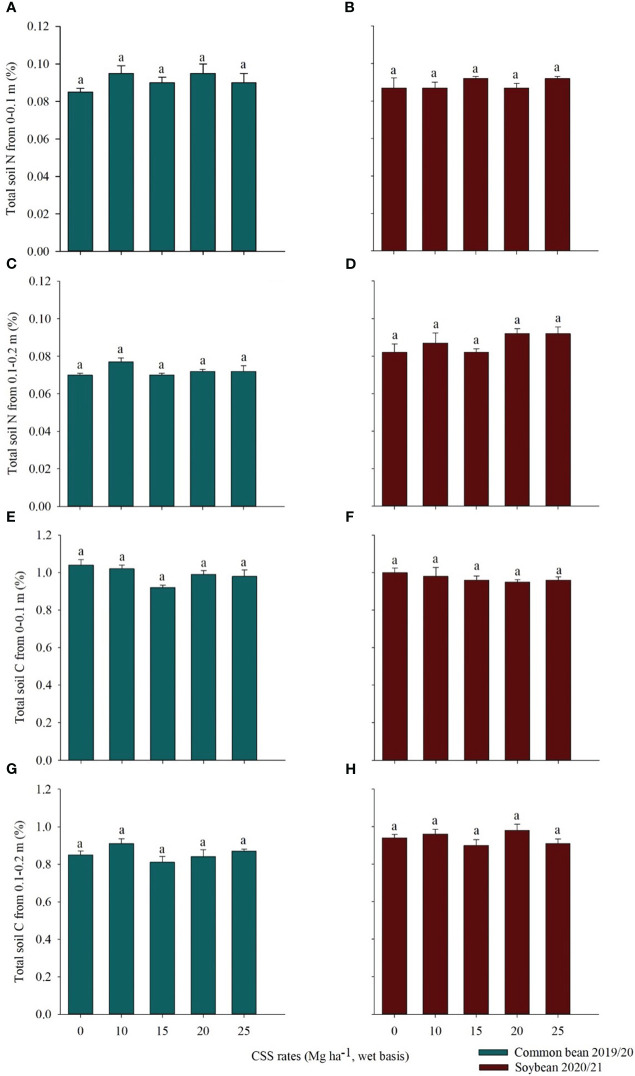
Total soil N from 0*–*0.1 m **(A, B)** and 0.1*–*0.2 m **(C, D)**, total soil C from 0*–*0.1 m **(E, F)** and 0.1*–*0.2 m **(G, H)** after common bean and soybean cultivation under the residual effect of the application of rates of composted sewage sludge (CSS). Means ± standard error followed by the same letter do not differ by Tukey test at 5% probability.

Total soil N and C were evaluated at two depths (0–0.1 m and 0.1–0.2 m) after the cultivation of each crop studied. It was possible to note that both total N and total C at each depth were not influenced by the residual effect of CSS for common bean and soybean (**Figure 5**). For common bean, total N at the 0-0.1 m depth ranged from 0.09 to 0.08% for the 10 Mg ha^-1^ CSS rate and for the control treatment, respectively (**Figure 5A**). At the depth of 0.1-0.2 m, the values were similar in all treatments, 0.07% (**Figure 5C**). Total C ranged from 1.04 to 0.92% at the 0*–*0.1 m depth, in the control treatment and at the 15 Mg ha^-1^ CSS rate, respectively (**Figure 5E**) and 0.91 to 0.81% at the 0.1*–*0.2 m depth, at the 10 and 15 Mg ha^-1^ CSS rates (**Figure 5G**).

In soybean, total soil N at the depth 0-0.1 m was similar in all treatments, with a value of 0.09% (**Figure 5B**). The same was observed for the 0.1*–*0.2 m depth (**Figure 5D**). The total C in the depth of 0*–*0.1 m varied from 1 to 0.95%, in the control treatment and in the rate of 20 Mg ha^-1^ CSS rate, respectively (**Figure 5F**). In the depth 0.1-0.2 m, the values found were similar in all treatments, 0.9% (**Figure 5H**).

### 3.3 Nodulation, enzymes, nitrate, and ammonium

**Figure 6:**
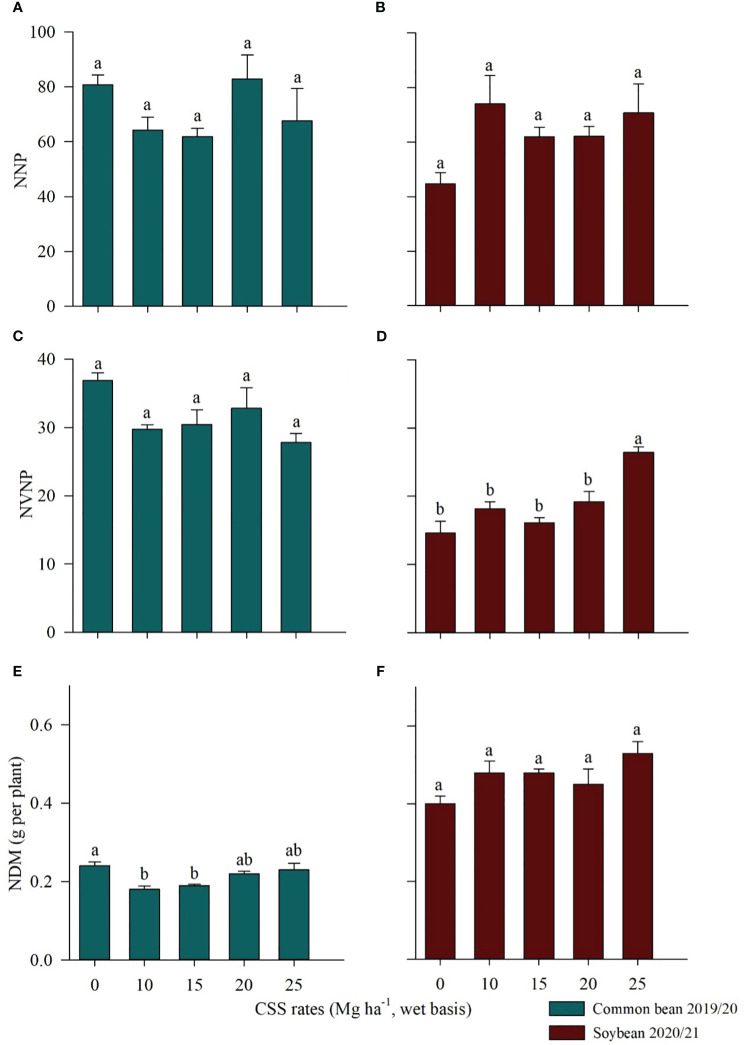
Number of nodules per plant (NNP) **(A, B)**, number of viable nodules per plant (NVNP) **(C, D)**, and nodule dry matter (NDM) **(E, F)** in common bean and soybean plants cultivated under the residual effect of the application of rates of composted sewage sludge (CSS). Means ± standard errors followed by the same letter did not differ from each other by Tukey’s test at 5% probability.

To evaluate the nodulation efficiency in common bean and soybean plants, the number of nodules per plant (NNP), the number of viable nodules per plant (NVNP), and the nodule dry matter (NDM) were determined (**Figure 6**). The NNP and NVNP in common bean (**Figures 6A, C**) and NNP and NDM in soybean (**Figures 6B, F**) did not differ between treatments as a function of the residual effect of the CSS rates evaluated. However, the values of NDM in common bean differed between the rates evaluated: control treatment and the 20 and 25 Mg ha^-1^ CSS rates presented the highest values, i.e., 0.24, 0.22, and 0.23 g per plant, respectively (**Figure 6E**). In soybean, the NVNP values differed between the rates evaluated: the residual of the highest rate of CSS (25 Mg ha^-1^ CSS) provided the largest number of viable nodules, with an average of 26 nodules per plant (**Figure 6D**).

**Figure 7:**
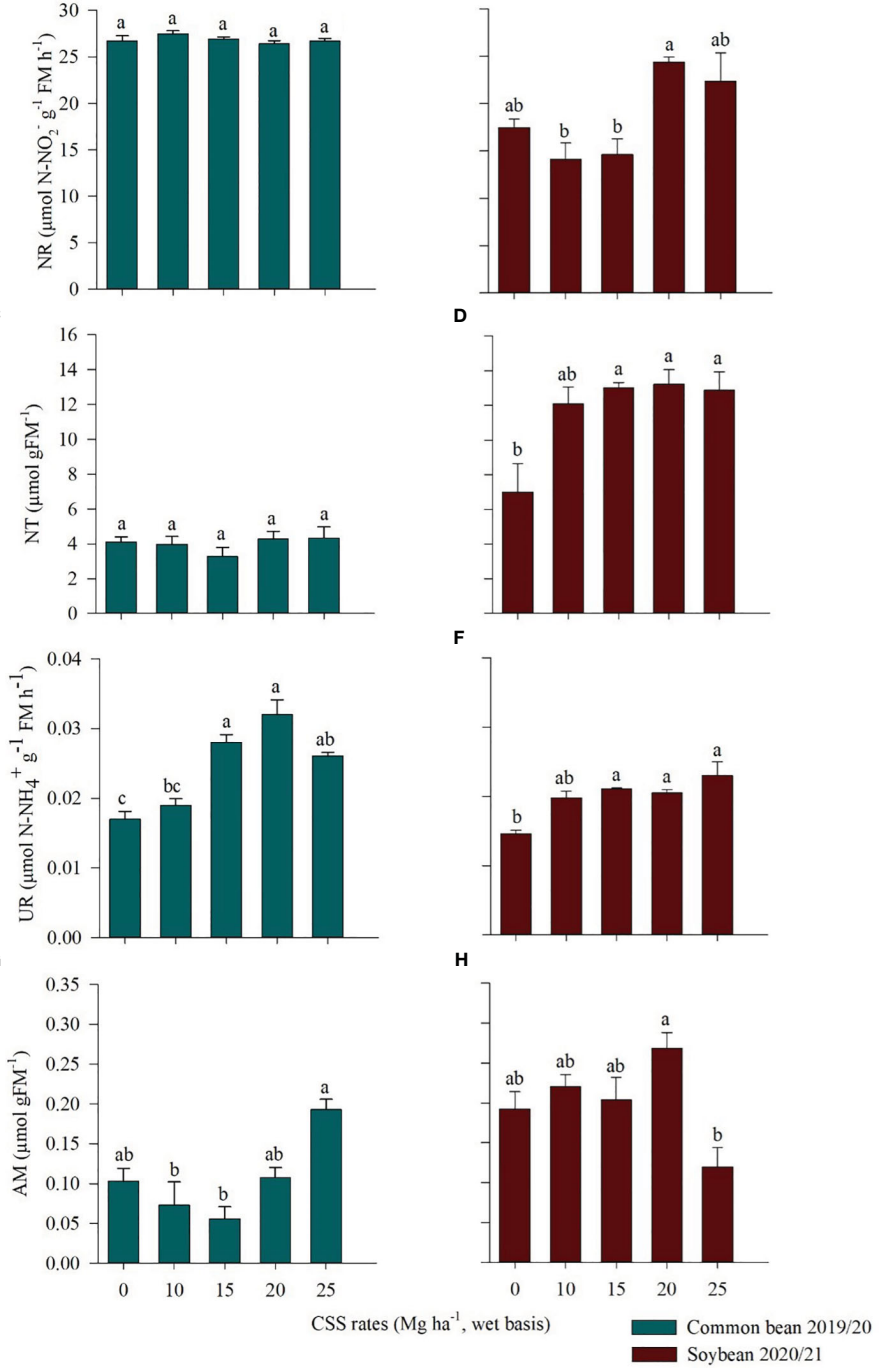
Nitrate reductase (NR) **(A, B)**, nitrate (NT) **(C, D)**, urease (UR) **(E, F)**, and ammonium (AM) **(G, H)** in common bean and soybean plants cultivated under the residual effect of the application of composted sewage sludge (CSS). Means ± standard errors followed by the same letter did not differ from each other by Tukey’s test at 5% probability.

The residual effect of CSS application affected the activity of nitrate reductase (NR) and the nitrate (NT) content in soybean (**Figures 7B, D**) and the urease activity (UR) and ammonium (AM) content in beans and soybeans (**Figures 7E–H**), respectively. In common bean, the highest values of UR activity were observed in the treatments that received 15 and 20 Mg ha^-1^ CSS (range 0.02 to 0.03 µmol N-NH_4_^+^ g^-1^ FM h^-1^) (**Figure 7E**). Consecutive application of the highest rate (25 Mg ha^-1^) of CSS promoted the greatest gains in AM (0.19 µmol g FM^-1^) (**Figure 7G**). There was no difference between the treatments tested in relation to the activity of the NR enzyme and the NT content in the common bean (**Figures 7A, C**).

In soybean, the 20 and 25 Mg ha^-1^ CSS rates increased NR activity, with values between 24.39 and 22.36 µmol N-NO_2_^-^ g^-1^ FM h^-1^, respectively (**Figure 7B**). Soybean NT content increased as the CSS rate increased compared to the control treatment, reaching the highest value at 20 Mg ha^-1^ (13.23 µmol g FM^-1^) (**Figure 7D**). In soybean, UR activity was also influenced by CSS, with an increase in activity in the presence of CSS (**Figure 7F**). The AM content was also affected by the residual effect of CSS: the highest value was found for the 20 Mg ha^-1^ CSS rate (0.20 µmol g FM^-1^) and the lowest for the of 25 Mg ha^-1^ CSS rate (0.09 µmol g FM^-1^) (**Figure 7H**).

### 3.4 Ureides, amino acids, and protein

**Figure 8:**
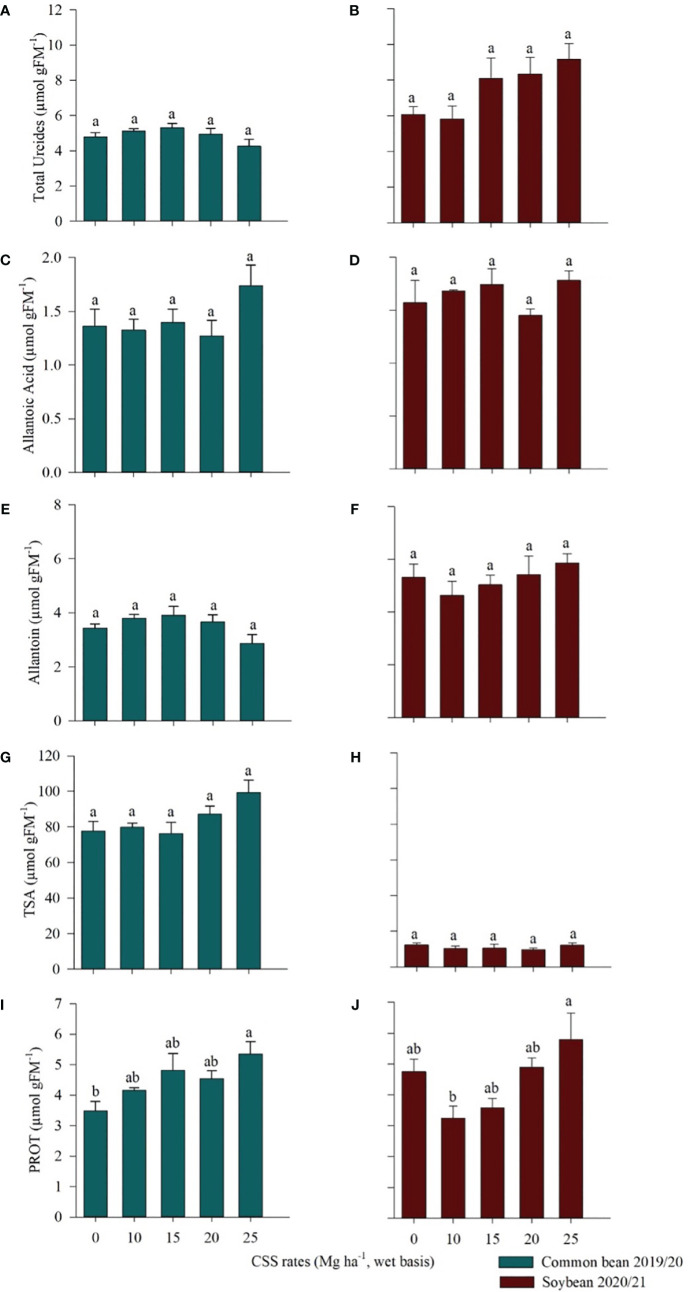
Total ureides **(A, B)**, allantoic acid **(C, D)**, allantoin **(E, F)**, total soluble amino acids (TSA) **(G, H)**, and protein (PROT) **(I, J)** in common bean and soybean cultivated under the residual effect of the application of rates of composted sewage sludge (CSS). Means ± standard errors followed by the same letter did not differ from each other by Tukey’s test at 5% probability.

There was no difference in ureides, allantoic acid, and allantoin among treatments in common bean and soybean experiments (**Figures 8A–F**). There was also no difference in total soluble amino acids (TSA) content among the treatments evaluated in bean and soybean experiments (**Figures 8G, H**). There was a difference regarding the proteins in the two crops. In common bean, the highest protein value (5.35 µmol g FM^-1^) was observed in the treatment of largest amount of CSS (25 Mg ha^-1^) (**Figure 8I**). The same behavior was observed in soybean, where the highest PROT content was found for the 25 Mg ha^-1^ CSS rate, i.e., 5.78 µmol g FM^-1^, which was different only from the treatment with 10 Mg ha^-1^ CSS (**Figure 8J**).

### 3.5 Production and productivity components

**Figure 9:**
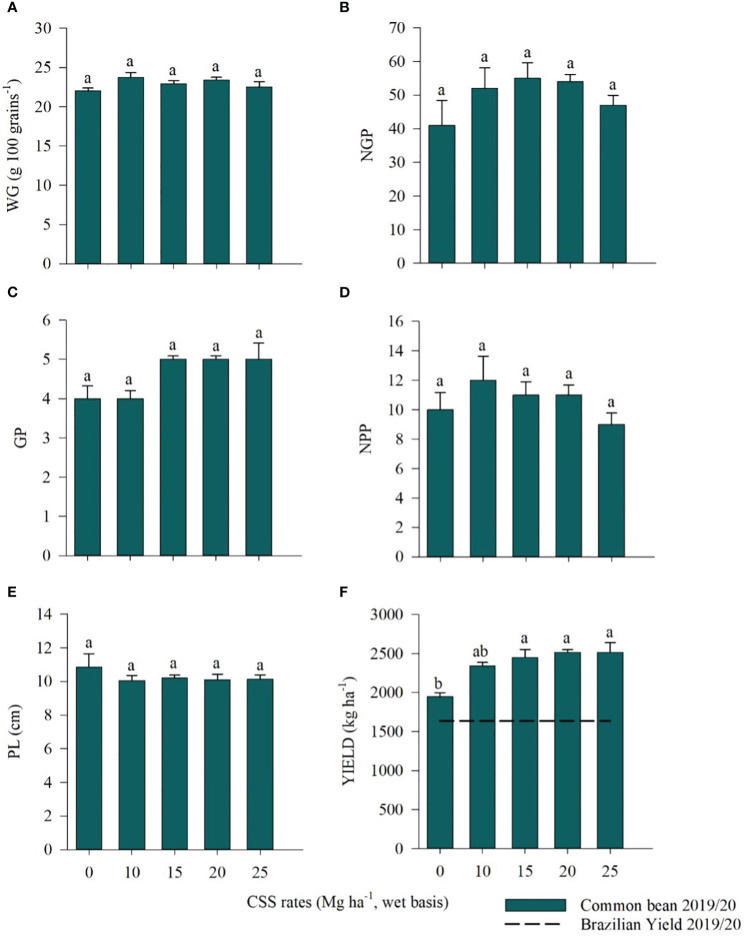
100-grain weight (WG) **(A)**, number of grains per plant (NGP) **(B)**, number of grains per pod (GP) **(C)**, number of pods per plant (NPP) **(D)**, pod length (PL) **(E)**, and yield **(F)** of common bean plants cultivated under the residual effect of the application of rates of composted sewage sludge (CSS). Means ± standard errors followed by the same letter did not differ from each other by Tukey’s test at 5% probability. Average productivity in Brazil (Conab, 2020).

For common bean, the 100-grain weight (WG), number of grains per plant (NGP), number of grains per pod (GP), number of pods per plant (NPP), pod length (PL), and yield were evaluated. There was no influence of the residual effect of CSS application for WG, NGP, GP, NPP and PL (**Figures 9A–E**). A difference in productivity was observed between the studied treatments in which there was a variation from 1945.27 kg ha^-1^ to 2515.72 kg ha^-1^, with the highest values found from the 15 Mg ha^-1^ CSS rate (**Figure 9F**).

**Figure 10:**
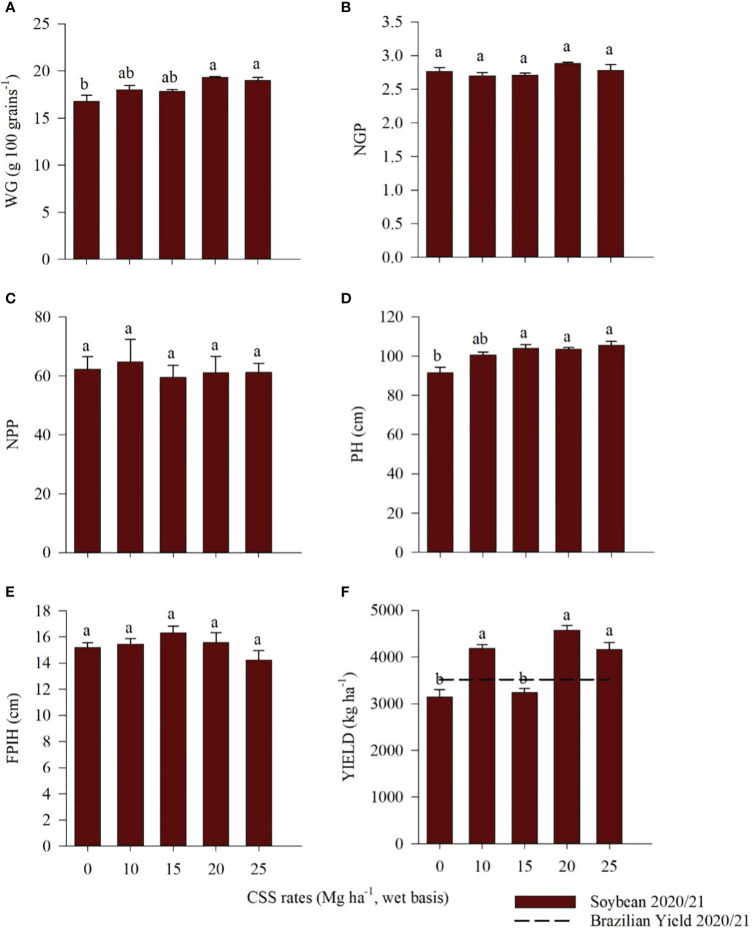
100-grain weight (WG) **(A)**, number of grains per pod (NGP) **(B)**, number of pods per plant (NPP) **(C)**, plant height (PH) **(D)**, first pod insertion height (FPIH) **(E)**, and yield **(F)** of soybean plants cultivated under the residual effect of the application of rates of composted sewage sludge (CSS). Means ± standard errors followed by the same letter did not differ from each other by Tukey’s test at 5% probability. Average productivity in Brazil (Conab, 2021).

For soybean, NGP, NPP and FPIH were not affected by the residual effect of CSS application (**Figures 10B, C, E**), while WG, PH and yield were influenced by the residual effect of CSS application (**Figures 10A, D, F**). The two highest rates (20 and 25 Mg ha^-1^ CSS) yielded 19.32 g and 18.97 g WG, respectively (**Figure 10A**), which implies that these grains had greater accumulation of photoassimilates. For PH, the three highest rates (15, 20, and 25 Mg ha^-1^ CSS) presented the highest values, with heights of 103.93, 103.51, and 105.52 cm, respectively (**Figure 10D**). In terms of productivity, the 20 Mg ha^-1^ CSS rate showed the highest productivity gain, with a value of 4574.51 kg ha^-1^, followed by 10 Mg ha^-1^ (4184.43 kg ha^-1^) and 25 Mg ha^-1^ (4162.63 kg ha^-1^) (**Figure 10F**).

### 3.6 Phytomass production and N accumulation in marandu palisadegra*ss*

**Figure 11:**
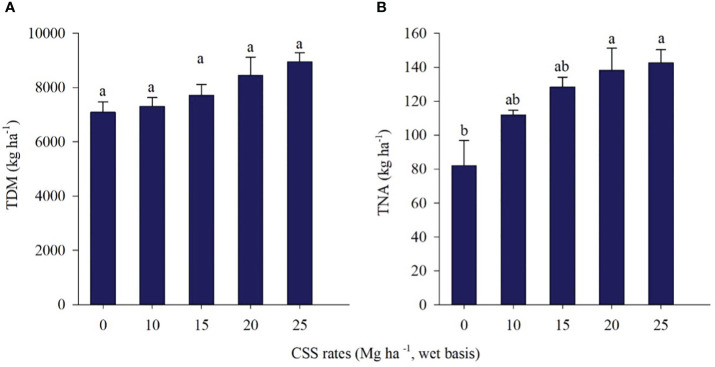
Total dry matter (TDM) **(A)** and total nitrogen accumulation (TNA) **(B)** in two cuts of marandu palisadegrass in the 2019/20 crop, cultivated under the residual effect of composted sewage sludge (CSS) application. Means ± standard errors followed by the same letter did not differ from each other by Tukey’s test at 5% probability.

**Figure 11** shows the total dry matter (TDM) and total N accumulation (TNA) in marandu grass after two cuts (70 and 140 DAE) in the 2019/20 crop year. The residual effect of CSS did not affect the TDM of marandu palisadegrass (**Figure 11A**), and the values ranged from 7090 kg ha^-1^ to 8947 kg ha^-1^, showing a 26% gain in TDM. On the other hand, TNA was influenced by the residual effect of successive applications of CSS, in which the control treatment differed from the treatments that received the highest rates of CSS (**Figure 11B**). CSS rates also increased N accumulation in marandu palisadegrass.

**Figure 12:**
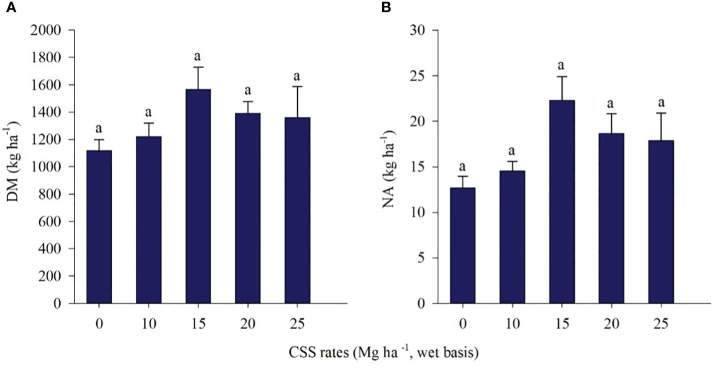
Dry matter (DM) **(A)** and nitrogen accumulation (NA) **(B)** of marandu palisadegrass in the 2020/21 crop year under the residual effect of composted sewage sludge (CSS) application. Means ± standard errors followed by the same letter did not differ from each other by Tukey’s test at 5% probability.

There was no effect of residual CSS application on dry matter (DM) and nitrogen accumulation (NA) in marandu palisadegrass at 60 DAE in crop year 2020/21 (**Figures 12A, B**). The values for DM ranged from 1118 kg ha^-1^ to 1567 kg ha^-1^, representing a 40% gain in DM. (**Figure 12A**). The accumulated amounts of N ranged from 13 kg ha^-1^ to 22 kg ha^-1^ of N per plant (**Figure 12B**).

## 4 Discussion

The FCI is an important parameter to indirectly evaluate the chlorophyll content and correlate it with the N levels in leaf tissue, since N is part of the molecular structure of chlorophyll (Filla et al., 2020). In common bean, there was an influence of the residual effect of the CSS rates, with the highest FCI value (38.18) found for the 10 Mg ha^-1^ CSS rate. These results differ from those found by Nakao (2015), who observed no influence of fertilization with organic compost on FCI of common bean, where the values ranged between 38.17 and 41.17. In soybean, there was no influence of the residual effect of CSS application, contrary to the study by Ragagnin et al. (2013), who tested different rates (1.0, 2.0, 4.0, and 8.0 Mg ha^-1^) using organic material from chicken litter and found higher chlorophyll contents compared to the control treatment.

The residual effect of successive CSS applications did not increase the leaf N content in common bean and soybean. However, these parameters remained within the appropriate range for both crops (30–50 g kg^-1^ for bean and 40–54 g kg^-1^ for soybean). This finding may be related to the higher content of potentially mineralizable N in the soil, which allows its release and use by crops throughout their development cycle, as occurs with common bean (Bettiol and Ghini, 2011). Boeira and Maximiliano (2009) reported that there is an effect of previous sludge applications on the N mineralization potential and that higher rates lead to the accumulation of NO_3_^-^ in the soil. It is known that soil nitrate can be easily lost by leaching. Therefore, adequate rates of CSS should always be applied to maintain crop nutrition and avoid possible environmental risks (Moretti et al., 2015; Rigby et al., 2016). In addition, crop rotation and the use of cover crops are essential so that part of leachable N is recovered through N-cycling (Borges et al., 2014).

The SDM, RDM, SNA, and RNA of soybean increased with the residual application of CSS. These results agree with other studies reporting that the residual effect of SS application provided the slow release of nutrients, in which the success of SS depends on the conditions of the production process, the rates used, the type of soil, and the crop cultivated (Melo et al., 2018). Yuruk and Bozkurt (2006) reported that SS treatments increased the grain yield and biomass of common bean and chickpeas (*Cicer arietinum* L.) and did not cause any nutrition imbalance of these plants.

Some studies have already shown that SS may favor nodulation in legume plants, such as soybeans and fava beans (Rebah et al., 2007; Eid et al., 2018). Furthermore, Rebah et al. (2007) indicated that several components of SS are essential to increase the activity of N-fixing bacteria, with nodulation notably favored when SS was used. In our study, CSS altered the dry matter of common bean nodules and increased the number of viable nodules in soybean with increasing rates of CSS. Several studies have shown that total nodule number and nodule dry matter analyses (Ferreira and Castro, 1995; Martins et al., 2022) are efficient for estimating BNF. Thus, in a study performed to evaluate BNF with the use of composted SS, there was an increase in the number of nodules up to a rate of 19 Mg ha^-1^ SS and an increase in dry matter at rates of 30 and 40 Mg ha^-1^ associated with seed inoculation (Lobo et al., 2012). In addition, the ^15^N natural abundance technique used in our study to accurately estimate how much of the fixed N comes from BNF, showed that values from 90 to 97% of the common bean N in the CSS treatments comes from BNF and the difference with the control treatment may have been due to the transformations of the N present in the compost in the soil.

It is noteworthy that the increase in viable soybean nodules and the stability of % BNF during the soybean reproductive stage in our study indicates an efficient BNF in the soybean crop that was not negatively affected by CSS, as already concluded by Souza et al. (2009), who found that SS application at rates of up to 6 Mg ha^-1^ does not negatively affect soybean nodulation in a two-year period. In an experiment carried out under field conditions, Currie et al. (2003) demonstrated that applying SS can increase the BNF rates in soybean. Although we observed that the % BNF in soybean (calculated at R8 grains phenological stage) receiving CSS decreased compared to the control, about 95% (on average) of the fixed N was from BNF in the CSS treatments.

The concentration of cellular nitrate is the main factor controlling the expression of NR because this enzyme reduces nitrate in the cellular environment. If the concentration of NO_3_^-^ decreases, the NR activity will consequently decrease or, with the addition of N-NO_3_^-^, a higher concentration of nitrate and NR activity is expected (Hirel and Krapp, 2020). In the present study, for common bean, this relationship remained stable and was not affected by CSS application. For soybean, this relationship was true because CSS influenced the nitrate content and consequently the NR activity in soybean leaves, where the highest rates of CSS showed higher means for these two parameters.

The CSS altered urease activity and consequently the ammonium content in common bean and soybean leaves. However, in soybean, the highest rate of CSS (25 Mg ha^-1^) showed the lowest mean ammonium content. Urease activity is greatly influenced according to the type of nitrogen fertilization used, since the plant can uptake N as urea due to specific transporters (Witte, 2011). In addition, urea is also generated within plants through arginine catabolism (protein degradation) and photorespiration, which generates NH_4_^+^ that will be incorporated by glutamine synthetase (Rennenberg et al., 2010; Witte, 2011). Urea in the plant cytosol is hydrolyzed by urease and releases ammonia/ammonium that is used for amino acids biosynthesis (Carter et al., 2009; Witte, 2011). As in our study, we did not use any source of urea, the increase in urease activity, the decrease in NH_4_^+^ concentration and the increase in soluble proteins are linked to urea biosynthesis in the plant, promoting a rapid cycling of these metabolites and without causing damage to the plant, as they indicate an efficient assimilation of N generated in the form of NH_4_^+^.

Moreover, the materials involved in sludge composting may influence this characteristic of urease and ammonium because N-containing substances such as proteins, amino acids, and nucleic acids present in the sludge can release N as ammonium in the soil (Sharma et al., 2017; Raheem et al., 2018). The process of protein decomposition occurs in several steps and releases several amino acids. These amino acids undergo a deamination process, releasing the amine group as ammonia, which in the soil reacts quickly with water to form ammonium, which plants can uptaken (Vieira, 2017). Thus, all these metabolic processes involving urease and ammonium seem to have been influenced by CSS, but studies demonstrating these effects are still scarce.

The residual effect of CSS application positively influenced the protein content of beans and soybeans. The increase in protein content may be the compensation found in this study to maintain N-fixation; we did not observe changes in ureide metabolism, as they are the main N-containing molecules used for N transportation from nodules in bean and soybean to other plant tissues for use in protein biosynthesis (Díaz-Leal et al., 2012; Hirel and Krapp, 2020).

The consecutive CSS applications increased the plant height and the weight of 100 soybean grains, with the highest values found at the highest rates applied (20 and 25 Mg ha^-1^ of CSS). The same was observed in a study that tested organic fertilization through chicken litter at 3, 6, and 9 Mg ha-1 rates in a Cambisol. For the development and production components of soybean, there was an increase in plant height and 1,000-grain weight, in addition to the first pod insertion height, the number of pods per plant, and the soybean grain yield (Carvalho et al., 2011).

Fertilizers based on biosolids improve crop productivity and increase the availability of ammonium (NH_4_^+^) and nitrate (NO_3_^-^) in the soil due to organic N mineralization (Price et al., 2015; Wang et al., 2017b; Wijesekara et al., 2017). The use of CSS in this study affected common bean and soybean yields and maintained N levels within the recommended ranges for the crops. For common beans, plants that received CSS showed higher yields than those cultivated in the control treatment, with 2515 kg ha^-1^ production at the highest compost rate. Only the 10 Mg ha^-1^ CSS rate yielded production similar to that of the control, but all the studied treatments obtained a yield above the national average (1636 kg ha^-1^) for the 2019/20 crop of the third bean crop, according to a survey from CONAB (2020). For soybean, the averages obtained for the 10, 20, and 25 Mg ha^-1^ CSS rates remained above the national average (3527 kg ha^-1^) for the 2020/21 crop, confirming the CONAB survey findings (2021).

Such responses were already evidenced by Santos et al. (2011), who observed a 65% increase in common bean yield using CSS derived from the treatment of effluents of the beverage and paper industry compared to control treatment (without compost). One study that aimed to evaluate changes in organic matter contents and N forms in sludge-corrected soils and in the growth of maize (*Zea mays* L.) and fava beans (*Vicia faba* L.) also showed that the residual effect of CSS promoted a greater response in the grain yield of fava beans (Elsalam et al., 2021). In addition, our soybean yield values are similar to those found (3222 kg ha^-1^) in a study in which soybean was fertilized with mineral N by applying a rate of 300 kg ha^-1^ of N in the form of urea (Kubar et al., 2021). In the present study, with the rates of CSS applied, soybean yielding ranged from 3240 to 4574 kg ha^-1^.

In the first evaluation season (2019/20) of marandu palisadegrass, CSS rates led to an increase in N, especially at the highest rates (138.19 and 142.6 kg ha^-1^). In the second crop evaluated (2020/21), there was no increase in dry matter and N in marandu palisadegrass. Long-term CSS application can promote the increase in available N levels in the soil by increasing the amounts uptake by marandu palisadegrass, since there is a greater accumulation of this nutrient, especially at the highest rates of CSS supplied. The higher availability of N is also associated with the residual effect of CSS in the soil, which acts as a source of organic matter, improving fertility and providing N to the system, as has already been demonstrated for fescue forage (*Festuca arundinacea* Schreb.) for 19 years through the application of biosolids (Cogger et al., 2013).

Vendruscolo et al. (2016) showed that residual SS application in the recovery of a degraded area and the management with brachiaria (*Urochloa decumbens*) influenced the chemical attributes, especially the P and K contents, of the soil over nine years of evaluation.

In addition, cropping systems with forages preceding the main crop showed positive effects on the physicochemical characteristics of the soil, culminating in increased bean productivity on *U. ruziziensis* straw (Sabundjian et al., 2013; Amaral et al., 2016) and higher soybean grain yield and nutrient cycling on *Urochloa brizantha* straw (Moraes et al., 2014; Tanaka et al., 2019). Another important effect of these grasses is the high release and cycling of nutrients, especially N (Borges et al., 2014), P, and K (Pacheco et al., 2011).

It is believed that in the second crop evaluated, the absence of a CSS residual effect on marandu palisadegrass may have occurred due to its shorter cultivation time in the soil compared to the previous crop and to the immobilization-mineralization relationship of N in the soil. The type of straw on the soil surface, such as that with a high C/N ratio, promotes an increase in the rate of N immobilization by soil microorganisms and influences crop management, especially nitrogen fertilization (Amaral et al., 2016). At the beginning of the NTS, as is the case in the present study, the immobilization of nutrients and organic matter in the soil tends to be greater than the mineralization; however, mineralization changes with time, which increases the mineralization rate (Torres et al., 2008). Regardless, the consolidation of NTS leads to a balance between the mineralization and immobilization processes (Teixeira et al., 2011), in addition to mitigating N losses from the system through cycling and immobilization in its phytomass (Lara Cabezas et al., 2004).

## 5 Conclusions

The residual effect of successive CSS applications as a source of N in the bean-palisade grass-soybean rotation under no-tillage in the *Cerrado* may meet the N needs of the crops studied, especially at the highest applied rates (20 and 25 Mg ha^- 1^ of CSS, wet basis). Through the two CSS applications in highly weathered tropical soil, a residual effect of this fertilizer was observed as increases in common bean and soybean grain yield and adequate foliar N concentration in the crops. The percent of nitrogen derived from BNF in the shoot of beans and grains of soybean decreased with increasing CSS rates but remained stable with increasing nodule dry matter in beans and increasing viable nodules in soybean. The protein content increased with the residual effect of CSS. The mineralization of N present in the CSS influenced the nitrate content and the activity of nitrate reductase and urease in soybean. The urease activity and the ammonium content in the crops were affected by the residual effect of the compost, which may have occurred due to urea metabolism in leaves. The residual effect of CSS application in *Cerrado* soil may meet the long-term nutritional N demand of marandu palisadegrass under NTS and promote greater N cycling to subsequent crops, leading to a balance between the immobilization and mineralization rates of N. In terms of sustainable agriculture, the use of sewage sludge as composted organic fertilizer (i.e., CSS) can help in mitigating problems (e.g., food insecurity) due to the lack of both: *i*) mineral fertilizers, available for commercialization in Brazilian agriculture; *ii*) decreasing fertile lands, in addition to; *iii*) reducing the difficulties in using mineral fertilizers, especially in the State of São Paulo. Furthermore, it is an appropriate final destination for sewage sludge, which continues to be produced on a large scale in Brazil.

## Data availability statement

The raw data supporting the conclusions of this article will be made available by the authors, without undue reservation.

## Author contributions

MB: Conceptualization, Methodology, Software, Writing – original draft. LS: Data curation, Investigation, Writing – review & editing. MT: Data curation, Investigation, Validation, Visualization, Writing – review & editing. LS: Formal Analysis, Investigation, Methodology, Writing – review & editing. AC: Data curation, Formal Analysis, Validation, Writing – review & editing. JL: Data curation, Formal Analysis, Validation, Writing – review & editing. CA: Validation, Writing – review & editing. ZH: Data curation, Writing – review & editing. FZ: Visualization, Writing – review & editing. AJ: Validation, Writing – original draft. GC: Validation, Writing – review & editing. TN: Conceptualization, Funding acquisition, Project administration, Supervision, Visualization, Writing – review & editing.
